# Pupil light reflex evoked by light-emitting diode and computer screen: Methodology and association with need for recovery in daily life

**DOI:** 10.1371/journal.pone.0197739

**Published:** 2018-06-13

**Authors:** Yang Wang, Adriana A. Zekveld, Dorothea Wendt, Thomas Lunner, Graham Naylor, Sophia E. Kramer

**Affiliations:** 1 Section Ear & Hearing, Dept. of Otolaryngology-Head and Neck Surgery, VU University Medical Center and Amsterdam Public Health Research Institute, Amsterdam, The Netherlands; 2 Eriksholm Research Centre, Oticon A/S, Snekkersten, Denmark; 3 Department of Behavioral Sciences and Learning, Linköping University, Linköping, Sweden; 4 Linnaeus Centre HEAD, The Swedish Institute for Disability Research, Linköping and Örebro Universities, Linköping, Sweden; 5 Technical University of Denmark, Department of Electrical Engineering, Lyngby, Denmark; 6 Medical Research Council/Chief Scientist Office Institute of Hearing Research—Scottish Section (Part of The University of Nottingham), Glasgow, United Kingdom; Tokai University, JAPAN

## Abstract

**Objectives:**

Pupil light reflex (PLR) has been widely used as a method for evaluating parasympathetic activity. The first aim of the present study is to develop a PLR measurement using a computer screen set-up and compare its results with the PLR generated by a more conventional setup using light-emitting diode (LED). The parasympathetic nervous system, which is known to control the ‘rest and digest’ response of the human body, is considered to be associated with daily life fatigue. However, only few studies have attempted to test the relationship between self-reported daily fatigue and physiological measurement of the parasympathetic nervous system. Therefore, the second aim of this study was to investigate the relationship between daily-life fatigue, assessed using the Need for Recovery scale, and parasympathetic activity, as indicated by the PLR parameters.

**Design:**

A pilot study was conducted first to develop a PLR measurement set-up using a computer screen. PLRs evoked by light stimuli with different characteristics were recorded to confirm the influence of light intensity, flash duration, and color on the PLRs evoked by the system. In the subsequent experimental study, we recorded the PLR of 25 adult participants to light flashes generated by the screen set-up as well as by a conventional LED set-up. PLR parameters relating to parasympathetic and sympathetic activity were calculated from the pupil responses. We tested the split-half reliability across two consecutive blocks of trials, and the relationships between the parameters of PLRs evoked by the two set-ups. Participants rated their need for recovery prior to the PLR recordings.

**Results:**

PLR parameters acquired in the screen and LED set-ups showed good reliability for amplitude related parameters. The PLRs evoked by both set-ups were consistent, but showed systematic differences in absolute values of all parameters. Additionally, higher need for recovery was associated with faster and larger constriction of the PLR.

**Conclusions:**

This study assessed the PLR generated by a computer screen and the PLR generated by a LED. The good reliability within set-ups and the consistency between the PLRs evoked by the set-ups indicate that both systems provides a valid way to evoke the PLR. A higher need for recovery was associated with faster and larger constricting PLRs, suggesting increased levels of parasympathetic nervous system activity in people experiencing higher levels of need for recovery on a daily basis.

## Introduction

### Physiological assessment of autonomic nervous system functioning

The autonomic nervous system (ANS) is crucial to the human body as it regulates both the internal environment and the sequence of basic physiological events allowing an organism to optimally adjust to environmental changes [1]. The ANS is divided into two branches: the sympathetic nervous system (SNS) and the parasympathetic nervous system (PNS). The SNS is known to govern the ‘fight or flight’ response, while the PNS is commonly considered to be in control of the ‘rest and digest’ response.

Several methods to measure ANS activity and the relative contribution of the SNS and PNS systems exist. For example, measuring heart-rate variability (HRV) provides information about the relative contribution of the SNS and PNS branches into the total ANS activity [2]. The low-frequency components of HRV (0.04–0.15 Hz) index sympathetic activity, while the high-frequency components (0.15–0.40 Hz) are associated with parasympathetic activity [2].

### The pupil size reflects SNS and PNS activation

Similar to cardiovascular responses such as HRV, changes in an individual’s pupil size are also under the direct control of the ANS. Both PNS and SNS activation are reflected in the diameter of the pupil [3]. Thus, measurement of the pupil diameter provides a way to assess both the SNS and the PNS. Activation of the SNS results in pupil *dilation*, via innervation of the dilator muscles, and activation of the PNS results in pupil *constriction*, via innervation of the sphincter (constrictor) muscles of the eye [3].

**Fig 1:**
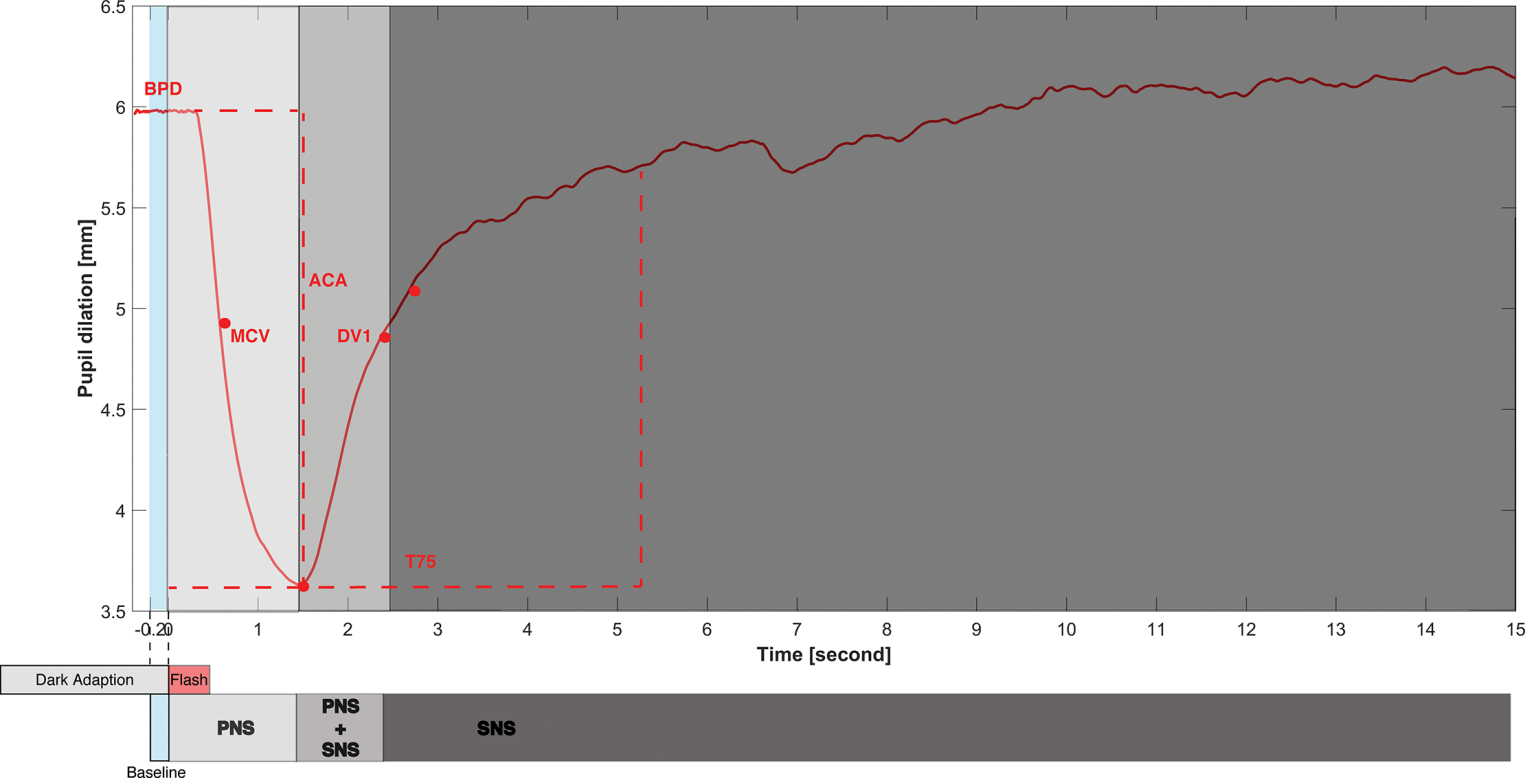
Schematic illustration of a pupil light reflex after the presentation of a flash light. BPD: baseline pupil diameter; MCV: maximum constriction velocity; ACA: absolute constriction amplitude; RCA: relative constriction amplitude; T75: time to reach 75% of initial resting diameter during pupillary re-dilation; DV1: Dilation velocity at 1s after Maximum constriction; PNS: contribution of parasympathetic nervous system; SNS: contribution of the sympathetic nervous system.

A measure that has been used to assess PNS activity is the pupil light reflex (PLR) [4]. The PLR is the rapid constriction of the pupil diameter in response to an increase in light intensity [5]. Light falling on the retina(s) leads to increased neural activity in the pretectal regions and stimulation of the Edinger-Westphal nucleus, where preganglionic parasympathetic neurons are activated and innervate the ciliary ganglion. These in turn command the constrictor muscles to tighten and this leads to pupil constriction. Both the ciliary ganglion and constrictor muscles contain receptors for Acetylcholine, which is the main neurotransmitter of the PNS [3, 4]. A typical PLR consists of three different phases (see different grey areas in Fig 1). It starts with a fast constriction phase shortly after the eye has been exposed to a light stimulus. Then an early fast re-dilation of the pupil diameter occurs, followed by a slow re-dilation phase during which the pupil diameter returns to its original size. According to Loewenfeld and Lowenstein [3], increased PNS activation is predominantly reflected in the fast constriction phase. The early fast re-dilation phase reflects both a reduction in PNS activation and an increase in SNS activation, while the later slower re-dilation phase is predominantly the result of innervations of the SNS system. PLR measurements have been widely used as a clinical diagnostic tool to assess PNS dysfunction. As summarized in a recent systematic review [4], the PLR can effectively detect PNS dysfunction in people with cholinergic diseases like Alzheimer’s Disease [6, 7], Parkinson’s Disease [8], diabetes [9], and psychiatric conditions like anxiety disorder [10].

Whereas the PLR has been shown to be a valid and useful method to assess PNS dysfunction, it is important to note that the PLR parameters (see Fig 1) are highly sensitive to the characteristics of the stimuli used to generate the PLR. For example, the magnitude of the reflex is influenced by the color and the duration of the light stimulus [11, 12, 13]. Given that in the PLR studies conducted so far a variety of stimuli have been used to elicit the PLR, direct comparisons of the reflexes evoked in different studies cannot be made. A standard method to elicit the PLR does not yet exist. Most PLR-related studies have used LED(s) as the light source to generate the flash evoking the PLR [4]. The LEDs are usually mounted either on a standalone device (e.g. A-1000, Neuroptics Inc, San Clemente, CA) or on a hardware interface [14], which can be relatively expensive and technically demanding. In contrast, computer screens are generally available, and have been suggested to be an effective light source to generate PLRs [15, 16]. Recent developments within the disciplines of psychology and neuroscience demonstrate that PLR is not merely a low-level reflex, but is also influenced by higher-level visual and cognitive processing, at least when relatively complex test paradigms and visual stimuli including context are used. For instance, researchers reported that PLR is sensitive to cognitive processing [15, 17, 18, 19] and visual attention [20, 21, 22, 23, 24, 25]. A recent study suggested that the attentional modulation of the PLR is related to the activity of frontal eye fields, a prefrontal cortical area governing the movements and attention of the eye [26, 27]. Most of these findings were obtained utilizing a computer screen to generate visual stimuli which is different from the traditional approach using LED. The visual stimuli generated by the computer screen in those studies were quite complex and involving higher-level context. The computer screen is also able to provide simple light stimuli like the light source required to elicit the PLR. As far as we know, there are no studies available that have directly compared PLRs generated using a computer screen to those generated with LED set-ups. Although a direct comparison could be difficult due to the different visual fields yielded by different light sources (light stimuli generated by LEDs have a narrow visual field, while a computer screen generates a wider field), we may still expect that parameters calculated from the PLRs evoked by a computer screen and those evoked by LEDs show high consistency (i.e. are associated) within subjects. The first aim of the present study was to examine the split-half test-retest reliability of the PLRs evoked using a computer screen and a LED device. We also aimed to compare the parameters of the PLRs evoked by these two methods.

### Fatigue and ANS functioning

Feeling fatigued is a common complaint that almost half of the adult population has experienced [28]. Fatigue is usually defined as a subjective feeling or mood state of tiredness [29]. Meijman and Schaufeli [30] defined fatigue as: *“*The change in the psychophysiological control mechanism that regulates task behaviour, resulting from preceding mental and/or physical efforts which have become burdensome to such an extent that the individual is no longer able to adequately meet the demands that the job requires on his or her mental functioning; or that the individual is able to meet these demands only at the cost of increased mental effort and coping with increased task resistance”.

Daily-life fatigue may emerge after frequent exposure to stress without adequate recovery from it afterwards, and this could be a major cause of societal problems like work productivity loss, sick leave, and even psychiatric disorders [31, 32, 33, 34, 35]. The most intuitive way to assess fatigue is via self-report questionnaires [29, 36, 37]. Several examples of self-report outcome measures assessing fatigue are available. One is the Profile of Mood States questionnaire, which includes assessment of fatigue and vigor from the perspective of mood state [38, 39]. The Chalder fatigue scale [40] is a questionnaire which assesses both mental and physical fatigue, and evidence suggests it to be an effective tool to evaluate daily fatigue in different languages and populations [41, 42]

There is evidence showing that fatigue may not only emerge from stressful events or activities, but also from the lack of ability to adequately recover from stress [31]. The early symptoms of fatigue can be measured by assessing an individual’s *need for recovery*. The concept of need for recovery reflects the ability to cope and recover from fatigue and distress [31]. Insufficient recovery after work is an intermediate stage between exposure to the stressful working situation and the development of long-term fatigue related health issues, like burnout [31, 33, 43]. The Need For Recovery (NfR) scale is an eleven-item scale including items like: ‘I find it difficult to relax at the end of working day’ or ‘In general, it takes me over an hour to feel fully recovered after work’ [31]. Multiple studies have confirmed the quality and validity of this scale for evaluating work-related need for recovery [31, 33, 44, 45]. The NfR scale will be used in the current study to evaluate need for recovery experienced in daily-life.

Fatigue also influences physiological measurements indexing ANS activity. For example, fatigue-induced ANS dysfunction has been found in patients with multiple sclerosis [46], chronic fatigue syndrome [47] and cancer [48, 49]. A study by Tanaka et al. [50] measured high-frequency and low-frequency HRV when exposing a group of healthy adults to a 2-back task. Results of a correlation analysis indicated that higher levels of self-reported daily-life fatigue, as measured by the Chalder fatigue scale [40], were positively correlated with the low-frequency (sympathetic) index and negatively correlated with the high-frequency (parasympathetic) index of HRV before the task. As a result, current evidence based on HRV indicates that increased levels of fatigue may be associated with higher levels of SNS and lower levels of PNS activity.

In a previous study, we investigated the relationship between self-reported need for recovery and the task-evoked pupil dilation response during a speech perception in noise task [51]. The task-evoked pupil dilation reflects the balance or ratio of sympathetic and parasympathetic activation [52, 53, 54]. In conditions of ambient illumination, the response consists of a ‘direct’ SNS dilation-component and an additional component that emerges because of a task-evoked reduction in PNS activity [55]. Importantly, in darkness, PNS activity is maximally inhibited, so PNS has minimal effect on the task-induced pupil dilation [55]. Therefore, in darkness, the dilation is mainly driven by SNS activation. Hence, presenting identical stimuli and tasks in dark and light conditions allows the identification of the relative contribution of PNS and SNS components to the pupil dilation response. This aids the understanding of the origin of pupillometry findings. Wang et al. [51] reports task-evoked pupil response data for 27 normally-hearing healthy adults performing a speech reception threshold test in light conditions. We observed that a higher NfR score was associated with a smaller task-evoked pupil dilation response. However, in the same session, these participants also performed the same task in darkness, whereupon we observed no relationship between NfR and pupil dilation (unpublished data). Hence, the findings may suggest that a higher need for recovery is associated with reduced inhibition of the parasympathetic component of the pupil dilation response, resulting in reduced pupil dilation in light but not in dark where the PNS contribution to pupil size is negligible. This would be consistent with the notion of the PNS being the ‘rest and digest’ branch, meaning that a higher need to rest is associated with increased PNS activation–or less inhibition of the PNS in order to cope with the fatigue [56]. However, this interpretation is contrary to the evidence provided by Tanaka et al. [50] who claimed that increased daily-life fatigue is associated with *reduced* PNS activation, as indicated by their HRV analysis. To test this association, the second aim of the current study was to investigate the associations between PLR parameters (reflecting PNS activity) and need for recovery in healthy adult individuals.

Thus, the hypotheses tested in the present study were as follows:

- H1A: PLR parameters generated by the computer screen and the LED show good test-retest reliability across two consecutive blocks of trials.
- H1B: PLR parameters generated by the computer screen and the LED are consistent across subjects, but different set-ups may result in systematic differences in absolute values.
- H2: Higher levels of need for recovery are associated with increased PNS activity as indicated by PLR parameters indexing PNS activation.

## Methods

### Pilot study

Previous studies suggested that the PLR is sensitive to different characteristics of the light stimuli, including *Color* [11, 12], *Light Intensity* [10, 57, 58], and *Duration* [59]. Therefore, we first conducted a pilot study to confirm the influence of light intensity, flash duration, and color on the PLRs evoked by a computer-screen set-up. Twenty-nine healthy participants were included in the pilot testing. The details of the pilot study design and results can be found in S1 Appendix of this paper. The data indicate that our computer screen PLR set-up is sensitive to the characteristics of different light stimuli and therefore considered to be a valid method to be used in the main study (below).

### Participants

Participants for the main study were twenty-five normal-hearing native Danish participants (13 females). They were recruited from the subject pool of the Eriksholm Research Centre, Snekkersten, Denmark. Their mean age was 50.4 years (SD = 11.9), ranging from 26 to 69 years old. All participants had a normal or corrected to normal vision and no neurological, psychiatric or ophthalmological problems were reported by any of the participants. All participants provided written informed consent and the study was approved by De Videnskabsetiske Komiteer (Journal number: H-1-2011-033).

### Need for recovery scale

The Need for Recovery (NfR) scale is an eleven-item scale from the Questionnaire on the Experience and Evaluation of Work, which assesses the experience of daily work situations [31, 60]. The NfR scale focuses on the evaluation of fatigue caused by work and the need for recovery afterwards. Examples of items included in this scale are: “I cannot really show any interest in other people when I have just come home myself”, or “When I get home from work, I need to be left in peace for a while”. Responses for each item are either ‘yes’ or ‘no’. The total NfR score is the number of ‘yes’ responses (except for item 4, where ‘no’ signals an unfavorable situation) divided by the total number of items, presented as a percentage (i.e. range 0–100). A higher NfR score corresponds to greater need for recovery reported by the respondent.

The NfR scale was originally designed and validated in Dutch. We translated the questionnaire following the guidelines described by Sousa and Rojjanasrirat [61]. The first translator translated the original Dutch NfR scale into Danish. A second translator (bilingual in both Dutch and Danish) then performed a blind back-translation of this initial translated version (Danish to Dutch) to compare the back-translated version with the original version. If there was any disagreement between the back-translated version and the original version, consensus was achieved between the two translators. Finally, a third native Danish person checked the language of the translated questionnaire and this resulted in the final version. The Danish NfR scale can be found in S2 Appendix of this paper.

### Pupillometry

We used an SMI RED 500 (SensoMotoric Instruments, Berlin, Germany) remote eye tracking system to record the pupil response of both eyes with 120 Hz sampling rate and a spatial resolution of 0.03°. Only data from the left eye were used in the analyses in the current study.

### Conditions and procedure

All participants visited the lab for a two-hour experiment. Participants were asked not to drink coffee on the same day before the experiment, as the effect of caffeine on the pupil response remains unclear [62, 63]. The NfR was completed by the participant shortly after he or she had arrived. During the test, participants were seated in a comfortable chair located in a dark sound-treated booth. The background luminance of the booth was less than 0.1 cd/m^2^. Head position was fixed using a chin-and-head rest. Light stimuli were presented either from a computer screen or a light-emitting diode (LED). We considered the light stimuli from these two light sources as two different PLR conditions.

For the computer screen condition, a DELL 1800FP (18.1") screen was used and placed in front of the participant. The computer screen was calibrated by a screen calibrator (X-Rite, i1 Display Pro) to ensure the uniformity of both the color and luminance of the light stimuli. The distance from the fixation mark (small white dot attached to the center of the screen) to the middle of the eyes was fixed to 55 cm, which yielded a visual angle of approximately 43°. Red light (peak wavelength: 680 nm) with 3 cd/m^2^ luminance was presented for 200 milliseconds (ms) from the full visual field of the screen.

For the LED condition, we used a set-up similar to that described by Steinhauer et al. [14]. A narrow angle (8°) red LED, placed 55 cm from the left eye, was used to generate the flash of light. The intensity of the light was calibrated to approximately 2 cd/m^2^. This intensity was determined based on feedback from the participants in the pilot study, which indicated that higher light intensity might cause discomfort to the participants. Four dim red LEDs, masked to pinhead size, were placed surrounding the flash LED in order to avoid the optokinetic effect (when a single light source in darkness starts to have apparent motion, it will evoke eye movements, and this may result in PLR artifacts). We used an Arduino UNO (http://arduino.cc) board, which is an open-source electronics prototyping platform based on user-friendly hardware and software, as the interface to control the onset and offset of the LEDs.

For each block, we first played an audio instruction asking the participants to get ready by putting their chin on the chin-rest. Then, after one minute dark adaptation time, flashes with 200 milliseconds duration were presented six times. The interval between each flash was 15 seconds, and pupil recording continued throughout the whole six trials (60 seconds dark adaption time plus 90 seconds testing time). Participants were requested not to blink during and a few seconds after each flash of light. In some cases, the pupillometry equipment had difficulty with recording the pupil size in completely dark conditions. A corrective action was then taken by the experimenter, reminding the participants to avoid eye-blinking or to keep their eyes open when necessary. Each condition consisted of one block of 6 practice trials and two blocks of 6 formal trials. The order of the conditions was randomized beforehand for each participant, such that half of the participants performed the SCREEN condition first, while the other half performed the LED condition first.

### Pupil data processing

The pupil data processing and analysis was performed using Matlab (MATLAB Release 2016a, The MathWorks, Inc., Natick, Massachusetts, United States). For each block, we discarded the first flash trial as it is usually associated with larger initial diameter and greater constriction compared to other trials due to a possible attention effect [14]. Baseline pupil diameter (BPD) was defined as the mean pupil diameter recorded during 200 ms before flash onset. Pupil diameters smaller than 0.001 mm together with zero diameter values were characterized as blinks. Trials in which the data included more than 10% blinks were rejected from the analysis. An additional blink detection procedure was applied to the pupil data between the start of the baseline to the maximum constriction part (where the pupil diameter was minimum); the threshold for the additional blink detection was set to 3%, such that traces with more than 3% blinks in this interval were omitted. Blinks that occurred in the re-dilation part of the trial were corrected by linear interpolation, blinks in the constriction part were corrected by applying a spline interpolation. We used a five-point moving average filter to smooth the data afterwards. The smoothed data then were time-aligned and averaged across all the accepted traces for a given condition, and the average BPD was subtracted to obtain the baseline-corrected pupil response.

Fig 1 shows a schematic illustration of the pupil response after a light flash, i.e. the PLR. It presents the parameters used in the analysis, as well as how the PNS and SNS contribute to different parts of the reflex. Besides the BPD, the following six PLR parameters were calculated: 1) the absolute constriction amplitude (ACA, mm), which is the magnitude of pupil constriction determined by the difference between BPD and the minimum pupil diameter during the constriction, 2) the relative constriction amplitude (RCA, %) describing the percentage of pupil constriction, determined by the ratio of ACA divided by BPD, 3) the maximum constriction velocity (MCV, mm/s), that is characterized by the largest first derivative of the pupil trace during the constriction, 4) the dilation velocity at 1 second (DV1, mm/s) is the slope of pupil diameter 1 s after the minimum pupil size, 5) and the latency to return to 75% (T75, s) of the initial BPD from the point of maximum pupillary constriction. These PLR parameters were selected based on the studies of Muppidi et al. [64], Steinhauer et al. [14], and Wang et al. [4]. The parameters reflect the innervation of parasympathetic and sympathetic nervous system during the reflex [3]. According to empirical evidence collected from the existing literature, the MCV, ACA and RCA are considered to indicate PNS activation, the BPD and DV1 reflect combined PNS and SNS activation, and the T75 is considered an indicator of SNS activation [4, 13, 14, 64].

### Statistical analyses

We first examined the descriptive statistics of the NfR data and the PLR parameters (MCV, ACA, RCA, BPD, DV1, T75), and tested the association between the PLR parameters separately for each set-up. Secondly, we tested the split-half test-retest reliability of the PLR measurements, again separately for each set-up. We compared the PLR parameters between the two consecutive blocks of trials of the same set-up by calculating Intraclass Correlations Coefficients (ICCs) for each PLR parameter. An ICC above 0.75 was considered as indicating absolute agreement between the two blocks [65]. Then, we ran dependent t-tests on the PLR parameters to test if there were any differences in the parameters of the PLRs evoked with the LED and the SCREEN. Next, we examined the Pearson correlation coefficients between corresponding PLR parameters obtained from the SCREEN and the LED conditions in order to test the correlations between the data acquired with the two set-ups. Finally, we tested the Spearman correlation coefficients between NfR, age and the PLR parameters obtained in both conditions. Non-parametric Spearman correlation coefficients were calculated as the NfR scores were not normally distributed.

## Results

**Fig 2:**
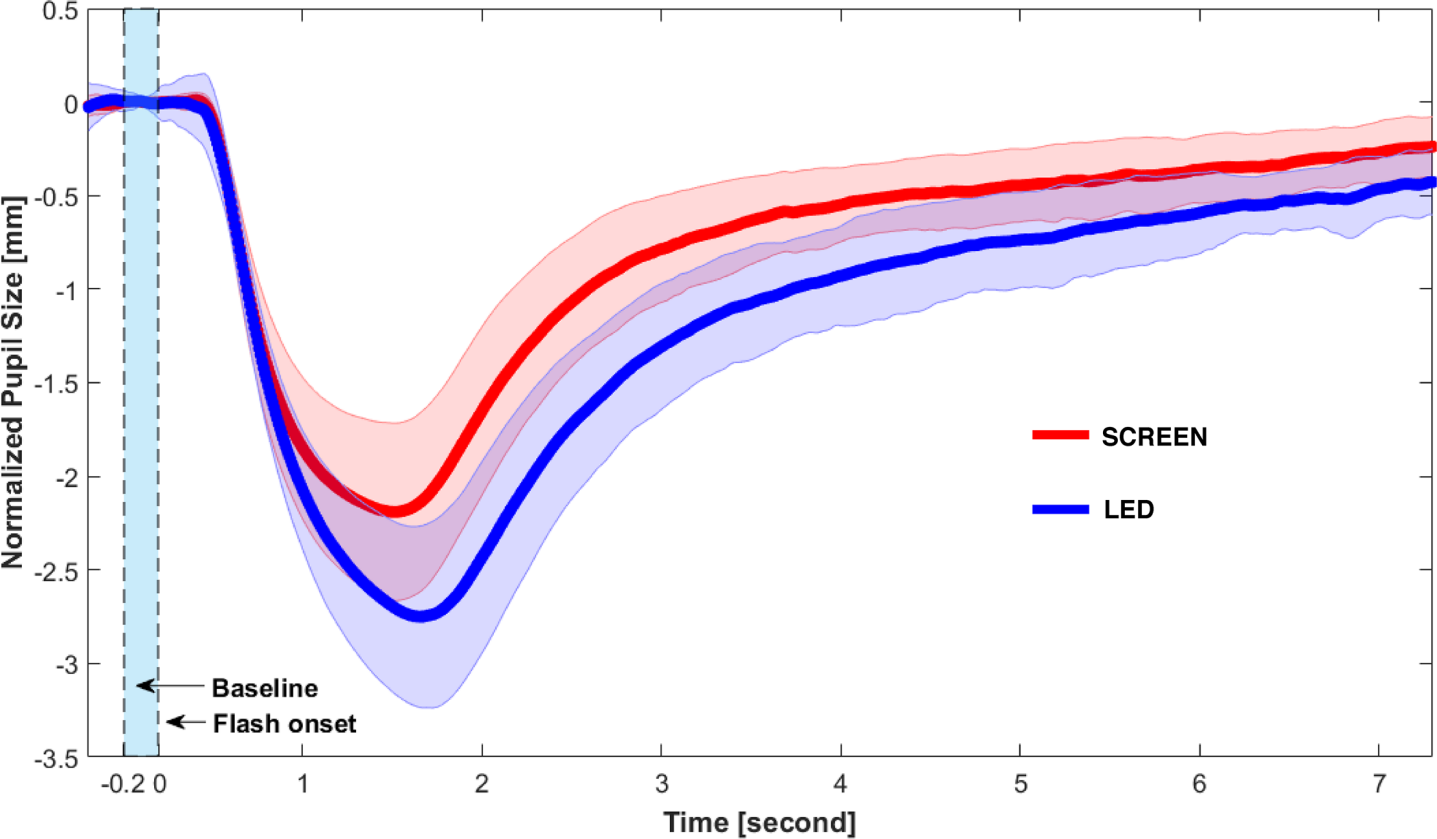
Baseline-corrected average PLRs of the SCREEN and LED conditions. Shaded area represents standard deviation.

The mean NfR score was 16% (SD = 14.4), and the distribution of the score was skewed to the right (skewness = 0.97). After pupil data preprocessing, 26 out of the 250 (25 participants x 10 trials) trials in the LED condition were discarded and 19 out of 250 trials in the SCREEN condition were discarded due to a large proportion of blinks (>10%). Descriptive statistics of the PLR data (for both the LED and SCREEN conditions) are provided in Table 1. The results are furthermore illustrated in Fig 2 in the form of the complete average traces.

**Table 1:** Descriptive statistics for the pupil light reflex parameters for the LED and SCREEN conditions.

PLR parameters	LED Mean (SD)	SCREEN Mean (SD)
MCV (mm/s)	5.80 (1.47)*	5.20 (0.91)*
ACA (mm)	2.74 (0.45)*	2.21 (0.46)*
RCA (%)	46.08 (3.93)*	41.79 (4.79)*
BPD (mm)	5.96 (0.85)*	5.27 (0.80)*
DV1 (mm/s)	4.52 (0.78)*	3.52 (0.79)*
T75 (s)	6.28 (2.18)	5.54 (2.30)

MCV: maximum constriction velocity; ACA: absolute constriction amplitude; RCA: relative constriction amplitude; BPD: baseline pupil diameter; DV1: Dilation velocity at 1s after Maximum constriction; T75: time to reach 75% of initial resting diameter during pupillary re-dilation

* p < 0.05 for paired-sample t-test of the difference between LED and SCREEN conditions

Table 2 shows the Pearson correlation coefficients between the PLR parameters for the LED and SCREEN conditions, respectively. The overall pattern of results is similar for the two set-ups, with relatively strong associations between ACA, MCV, BPD and DV1.

**Table 2:** PLR parameter correlations coefficients within LED and SCREEN condition.

**LED**						
	ACA	RCA	BPD	DV1		T75
MCV	0.47	-0.16	0.67*	0.32		-0.22
ACA		0.52*	0.84*	0.97*		0.09
RCA			-0.02	0.55*		0.06
BPD				0.79*		0.06
**SCREEN**						
	ACA	RCA	BPD	DV1		T75
MCV	0.82*	0.50	0.77*	0.80*		0.09
ACA		0.75v	0.84*	0.98*		0.21
RCA			0.28	0.78*		0.37
BPD				0.78*		0.03

MCV: maximum constriction velocity; ACA: absolute constriction amplitude; RCA: relative constriction amplitude; BPD: baseline pupil diameter; DV1: Dilation velocity at 1s after Maximum constriction; T75: time to reach 75% of initial resting diameter during pupillary re-dilation.

* Correlation is significant at the 0.0083 level (2-tailed).

Next, we ran a factor (principal component) analysis on these 6 parameters to examine the underlying latent factor structure. Results of varimax-rotated factor loadings are listed in Table 3. Both LED and SCREEN conditions ended up with two factors that had eigenvalue higher than 1. In both conditions, the first factor was mainly composed by MCV, ACA, BPD and DV1, and accounted for 51.51% (LED) / 61.43 (SCREEN) of the variance. Factor 2 was the combination of RCA and T75, and accounted for 29.10% (LED) / 24.57% (SCREEN) of the total variance. The calculated factor scores were stored and used later in the correlation analysis to test their correlations with NfR.

**Table 3:** Rotated factor loadings (varimax normalized) for each of PLR parameters (LED and SCREEN) along with their groupings within the two emergent factors.

**LED**		
	Factor 1	Factor 2
MCV	0.74	-0.53
ACA	0.93	0.36
RCA	0.23	0.78
BPD	0.95	-0.09
DV1	0.86	0.48
T75	-0.03	0.70
**SCREEN**		
	Factor 1	Factor 2
MCV	0.90	0.07
ACA	0.95	0.30
RCA	0.56	0.67
BPD	0.91	-0.10
DV1	0.92	0.35
T75	-0.04	0.90

MCV: maximum constriction velocity; ACA: absolute constriction amplitude; RCA: relative constriction amplitude; BPD: baseline pupil diameter; DV1: Dilation velocity at 1s after Maximum constriction; T75: time to reach 75% of initial resting diameter during pupillary re-dilation

The results of the ICC analysis for the split-half test-retest reliability analysis are shown in Table 4 for each of the two conditions. The ICCs were calculated with a two-way random-effect, single measures model [65]. ACA, RCA and BPD had ICC values higher than 0.75. The ICC values of the DV1 parameter of the PLR evoked in the SCREEN and LED conditions were 0.74 and 0.67 respectively. This is slightly lower than the threshold for good reliability. MCV and T75 showed lower ICC values, indicative of poor reliability.

**Table 4:** ICCs (single-measure) of the split-half test-retest reliability analysis (within each condition).

PLR parameters (95% CI)	MCV	ACA	RCA	BPD	DV1	T75
SCREEN	0.51 (0.14–0.76)	0.79* (0.58–0.90)	0.84* (0.66–0.93)	0.84* (0.61–0.93)	0.74 (0.49–0.88)	0.08 (-0.30–0.45)
LED	0.32 (0.09–0.63)	0.75* (0.51–0.88)	0.76* (0.48–0.89)	0.89* (0.78–0.95)	0.67 (0.39–0.84)	0.14 (-0.28–0.51)

ICC: Intraclass Correlation Coefficient; MCV: maximum constriction velocity; ACA: absolute constriction amplitude; RCA: relative constriction amplitude; BPD: baseline pupil diameter; DV1: Dilation velocity at 1s after Maximum constriction; T75: time to reach 75% of initial resting diameter during pupillary re-dilation

* ICC single measures value above 0.75

Paired-samples t-tests revealed significantly larger PLR parameters in the LED as compared to the SCREEN condition. This was true for the PNS parameters: MCV (t (24) = 2.77, p = 0.011), ACA (t (24) = 9.16, p < 0.001) and RCA (t (24) = 8.04, p < 0.001); and the combined PNS-SNS indicators: BPD (t (24) = 6.15, p < 0.001), DV1 (t (24) = 9.68, p < 0.001); but not for the SNS indicator T75 (t (24) = 1.79, p = 0.085). The LED condition induced bigger and faster pupil constrictions and initial redilation than the SCREEN condition.

The Pearson correlation coefficients between the corresponding PLR parameters generated by the LED and SCREEN conditions are shown in Table 5. Most of the corresponding PLR parameters obtained in different conditions (SCREEN versus LED) were significantly and positively associated with each other (except for T75), indicating high consistency between the two conditions.

**Table 5:** Pearson correlation coefficients between the parameters of the PLRs evoked by the SCREEN and LED conditions.

	SCREEN					
	MCV	ACA	RCA	BPD	DV1	T75
LED	0.69*	0.80*	0.83*	0.77*	0.74*	0.31

MCV: maximum constriction velocity; ACA: absolute constriction amplitude; RCA: relative constriction amplitude; BPD: baseline pupil diameter; DV1: Dilation velocity at 1s after Maximum constriction; T75: time to reach 75% of initial resting diameter during pupillary re-dilation.

* Correlation is significant at the 0.0083 level (2-tailed).

### Correlation analysis between NfR, age and PLR parameters

**Fig 3:**
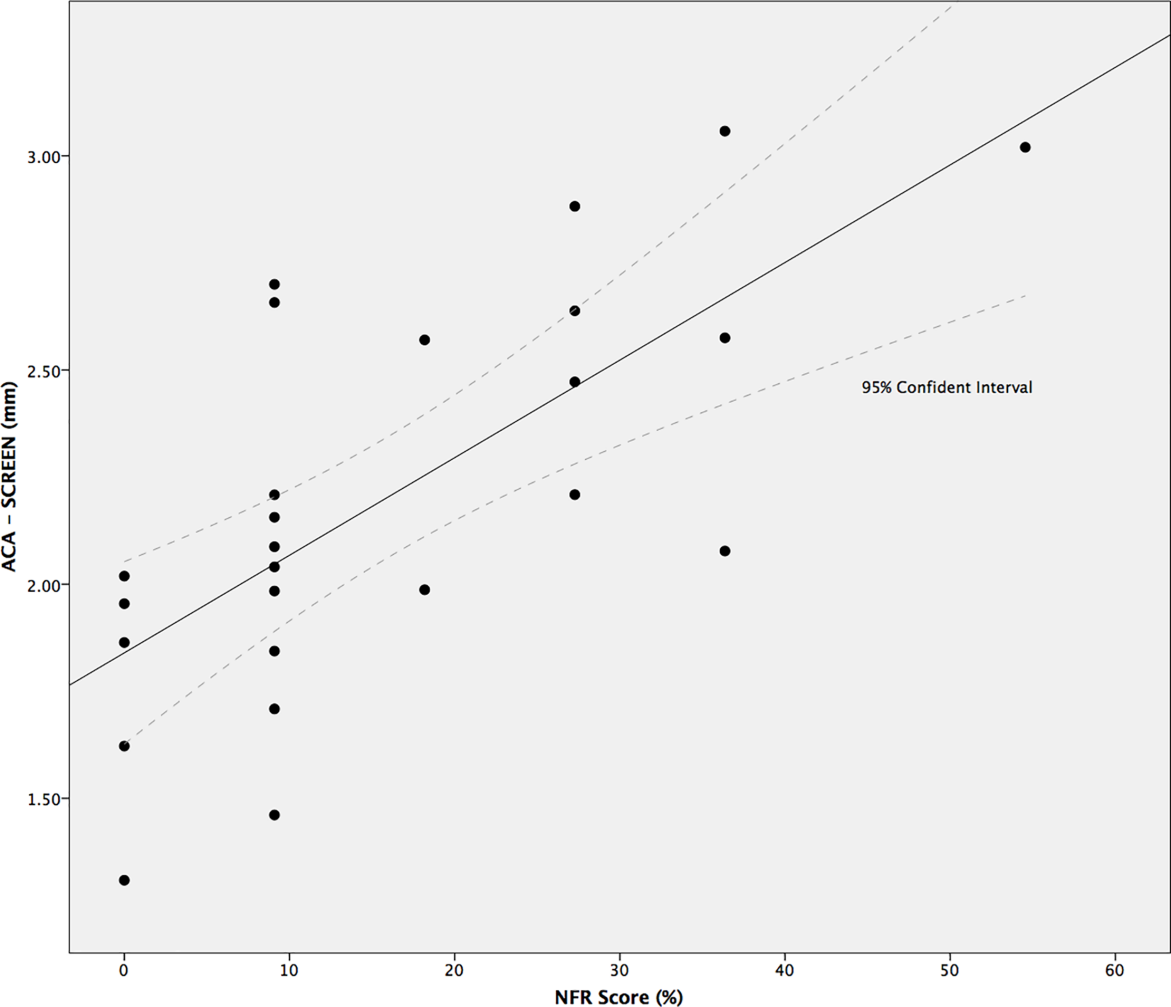
Scatter plot of NfR score against ACA (mm) from the SCREEN condition. Solid line represents the line of best fit; dashed line represents the 95% confident interval.

Because NfR scores were not normally distributed, we performed a non-parametric Spearman correlation analysis on the NfR scores and PLR parameters (MCV, ACA, RCA, BPD, DV1, T75) generated by both the LED and SCREEN conditions. A Holm-Bonferroni-correction was applied to correct for the multiple comparison. Table 6 shows the results. There were significant positive correlations between NfR and the ACA as assessed in both the LED (r (23) = 0.54, p < 0.005) and the SCREEN (r (23) = 0.71, p < 0.001) conditions, such that higher NfR scores were associated with larger pupil constriction. Similarly, we also found significant positive correlations between NfR and MCV, NfR and BPD, NfR and DV1 in both the LED (r (23) = 0.63, p < 0.002 for MCV; r (23) = 0.62, p < 0.002 for BPD; r (23) = 0.51, p < 0.01 for DV1) and SCREEN (r (23) = 0.62, p < 0.001 for MCV; r (23) = 0.68, p < 0.001 for BPD; r (23) = 0.69, p < 0.001 for DV1) conditions. These correlations indicate that higher levels of NfR were associated with faster and larger constriction, as well as a larger baseline and faster initial redilation velocity in the pupil light reflex. Fig 3 shows the scatterplot between the ACA measured in the SCREEN condition and the NfR scores. For both set-ups, we failed to find any significant correlations between NfR and the RCA and T75 parameters of the PLR. In addition, we separately tested the associations between the factor scores resulting from the factor analysis, and NfR. Results indicated that Factor 1, from both LED and SCREEN conditions, showed strong positive correlations with NfR (r (23) = 0.69, p < 0.001 for LED; r (23) = 0.76, p < 0.001 for SCREEN). In contrast, Factor 2 scores were not associated with NfR (r (23) = 0.05, p = 0.82 for LED; r (23) = 0.13, p = 0.55 for SCREEN).

**Table 6:** Spearman correlation coefficients between NfR score and PLR parameters.

	LED								SCREEN							
	MCV (mm/s)	ACA (mm)	RCA (%)	BPD (mm)	DV1 (mm/s)	T75 (s)	Factor 1	Factor 2	MCV (mm/s)	ACA (mm)	RCA (%)	BPD (mm)	DV1 (mm/s)	T75 (s)	Factor 1	Factor 2
NfR	0.58*	0.54*	0.26	0.62*	0.51*	0.42	0.69*	**0.05**	0.62*	0.71*	0.39	0.68*	0.69*	0.10	0.76*	0.13

NfR: Need for Recovery score (range 0–100%); MCV: maximum constriction velocity; ACA: absolute constriction amplitude; RCA: relative constriction amplitude; BPD: baseline pupil diameter; DV1: Dilation velocity at 1s after Maximum constriction; T75: time to reach 75% of initial resting diameter during pupillary re-dilation.

* Correlation is significant after Holm-Bonferroni correction; p < 0.008 for the first rank, p < 0.025 for the last rank

## Discussion

In the current study, we first aimed to develop a method to generate the PLR using a computer screen set-up and compare this method with the more commonly used LED system. The test-retest reliability of both set-ups were examined and we demonstrated that the computer screen set-up allows a sensitive and reliable registration of the PLR, whose reliability is similar to that of the PLR generated by a LED set-up. Our second aim was to examine associations between PLR parameters and a self-report measure of need for recovery, which was assumed to be associated with PNS related activity. Higher levels of need for recovery were associated with higher levels of PNS activity as reflected by PNS related PLR parameters in healthy individuals.

The pilot study described in S1 Appendix will inform future studies aiming to implement the measurement of the PLR using a computer screen. In S1 Appendix, we report how the PLR as measured with a computer screen set-up responds to light stimuli characterized by different colors, light intensities and durations. The findings of the pilot and current study indicate that it is critical to use the same set-up in studies focusing on the PLR and not switch between systems if one wants to compare absolute PLR values between studies or experiments. Also, the color of the light signal as well as the intensity and duration have an influence on the magnitude of most of the PLR parameters obtained. Our previous systematic review study [4] pointed out a lack of standardization in the assessment of PNS activity using PLR measurements. The current study contributes to the further standardization of a valuable method to evaluate PNS activity.

### PLR with screen and LED

In the current study, we evoked PLRs using a computer screen or a LED set-up. We observed relatively strong associations between ACA, MCV, BPD and DV1. It is generally assumed that some of the PLR parameters are relatively pure indicators of PNS activation (see Fig 1 and [4]), whereas others reflect the combined activation of the PNS and SNS [3]. DV1 is one parameter assumed to reflect both SNS and PNS [64], but the currently observed high (r = .97 for the LED and r = .98 SCREEN set-ups) correlation coefficient with the PNS index ACA may suggest that this redilation velocity parameter might also largely reflect PNS activation. The same may be true for BPD, albeit to a lesser extent. The relationship between BPD and ACA may also stem from the fact that the pupil size at maximum constriction was, on average, very small. For larger baseline pupil diameters, there is room for more constriction [66]. Results of the factor (principal component) analysis indicate that two distinct factors were underlying the PLR parameters. The first factor was mainly composed by MCV, ACA, BPD and DV1, and partly by RCA, which was likely related to PNS activity as discussed above. The second factor was made up by T75 and RCA, and might be associated with the SNS component. Contrary to the traditional view of RCA being a pure PNS indicator [4], the current finding may suggest that RCA was under the influence of both PNS and SNS. However, we have to admit the complexity of the PLR, as there is ample evidence suggesting that PLR could be affected by cognitive processing [15, 17, 18] and visual attention [20, 21, 22, 23, 24, 25]. As such, the PLR parameters may not reflect solely autonomic nervous system processes, but could involve complex higher-level processing.

To examine the test-retest reliability across trials within each of the two set-ups, we performed a split-half reliability (ICC) analysis on the PLR parameters determined in two consecutive blocks of trials within the same set-up. The ACA, RCA and BPD parameters showed good reliability, DV1 showed moderate reliability, and MCV and T75 showed relatively poor reliability across trials according to the results of ICC analysis. With increasing interest in PLR study, especially within the fields of psychology and neuroscience, extra caution needs to be taken considering the validity and reliability of these parameters. In Bär et al. [67], the test-retest reliabilities of their PLR measures (BPD, RCA, constriction velocity, redilation velocity and one-third-redilation recovery time) were tested over a time window of 10 minutes and again after one week. Results of an ICC analysis indicated good reliabilities for those parameters after one week. However, the data of the session 10 minutes after the initial test session indicated significant differences in constriction velocity between these two sessions. Thus, their results together with our current findings might indicate that the reliability of PLR parameters, like MCV and T75, might be better when assessing across a longer time span than across a short time span. This may suggest that these parameters are relatively vulnerable for adaptation effects when measuring the PLR repeatedly over a short time window. A retest session for both set-ups after one week would have been helpful to better evaluate the reliability of our SCREEN and LED set-ups. Note that we cannot perform such an analysis comparing the parameters of the PLRs evoked by the RL200 stimuli in the pilot and experimental study as the procedures (and hence, the resulting PLRs) differed substantially by including the warning signal in the pilot study. The parameters with good reliability (ACA, RCA and BPD) were all directly determined by the amplitude of the pupil signal. The other parameters (i.e., MCV, DV1s) were calculated from the derivative of the pupil signal, which may increase the risk of errors during the calculation as compared to the amplitude-determined parameters. Future researchers might consider examining the pupil curves manually, as it could be helpful to spot the potential errors. Evidently, in any case, one must take care to prevent the introduction of bias when selecting pupil traces manually.

As far as we know, this study is the first to compare the PLRs generated by computer screens with those evoked by a LED set-up in the same population. We found significant differences in the parameters of the PLRs evoked by the LED and SCREEN set-ups. The results indicate a systematic difference between the two methods, as all values were significantly larger in the LED set-up compared to the SCREEN set-up. Also, across conditions, the Pearson correlations of the corresponding PLR parameters were significantly positively associated with each other. This systematic difference was expected as previous studies [68] have shown that the PLR is very sensitive to stimulus characteristics such as the angle of the visual field generating the flash (the main difference between the SCREEN and LED set-up). The light stimuli generated by LED provided more concentrated light due to the narrow visual field as compared to the SCREEN set-up.

### Need for recovery and PNS activity

NfR was significantly associated with several PLR parameters from the SCREEN and LED conditions reflecting PNS activity or the combined activity of the parasympathetic and sympathetic nervous systems. A higher NfR was associated with a larger baseline pupil size, a larger constriction amplitude and a larger dilation velocity at 1 s after constriction. The association between NfR and ACA/MCV suggests that increased NfR is related to a more active PNS system, as ACA and MCV are considered as uncontaminated indices for PNS [8, 64]. It turns out that Factor 1 (mainly composed by MCV, ACA, BPD and DV1) is associated with NfR. As described above, Factor 1 is considered to be mainly related to PNS activity. Given the high associations between MCV, ACA, BPD and DV1 (see Table 6), we suggest that in the current study, MCV, ACA, BPD and DV1 were mainly reflecting PNS activity as well. Also, as we failed to find any correlation between NfR and either T75 or Factor 2 from the factor analysis (both of which are considered to reflect SNS [64] activity only), it seems likely that the correlations currently observed are driven by PNS activation rather than SNS activation. The current results are consistent with the previously observed relationship between increased levels of NfR and reduced task-induced pupil dilations in light conditions [51], which was interpreted as reflecting reduced inhibition of PNS activity. Reduced inhibition of the PNS activity is consistent with increased PNS activation.

The significant correlations between NfR and PLR parameters MCV, BPD and DV1 were observed in both the LED and SCREEN conditions. Among all the previously identified PNS-related PLR parameters (see Fig 1), RCA was the only one that did not show a significant correlation with NfR. Note that the NfR scores observed in the current study were relatively low and within a narrow range, as compared to previous data from similar or larger populations [33, 51]. However, this did not prevent us from finding significant correlations between the NfR and PLR parameters. It would be reasonable to expect these correlations to remain in populations with a larger variation in NfR.

To the best of our knowledge, there is only one other study reporting on the relationship between daily fatigue and PNS activity in healthy individuals. Tanaka and colleagues (2011) found a negative correlation between high-frequency power of HRV and subjective rating of daily fatigue, indicating reduced PNS activity was associated with higher level of daily fatigue, which is opposite to our present findings and those of [51]. Since the exact mechanism underlying the interaction between fatigue / need for recovery and PNS functioning is not yet clear, there are three possible explanations for the inconsistency between the present and Tanaka’s findings:

1) Although PNS activity measured by HRV is generally considered to be consistent with PNS activity measured by the PLR parameters ACA and MCV [4, 69], it is still possible that the cardiovascular indicators were not assessing exactly the same dimension of the ANS as the pupil indicators [70, 71]. This is pointed out by Janig and Habler [1] as they stated assessing autonomic functioning without distinction between different effector organs could lead to invalid conclusions. As far as we know, few studies have attempted to assess the association between PLR and HRV within normal populations. Among these studies, Bär et al. [70] measured HRV before a PLR test, and they observed a significant correlation between HRV complexity and latency of the PLR (which was not included in the current study) in healthy adults. Daluwatte et al. [71] measured simultaneous HRV before, during, and after the PLR testing phases in healthy children (8 to 16 years old). Results indicated no correlations between PLR and HRV parameters. The limited and contradictory evidence available makes it impossible to confirm that PLR and HRV are measuring the same dimension of ANS.

2) Different populations were assessed by the two studies. The participants in our study were Danish, whereas Tanaka’s participants were Japanese with slightly younger age (mean age, 43.6). Therefore, the inconsistency may also be due to the differences between the two samples.

3) The current paper focuses on NfR, whereas Tanaka et al. (2011) assessed task-induced fatigue by using the Chalder fatigue scale [40]. This may not correspond to daily-life NfR (a precursor of long-term fatigue). Having said that, the only study to measure the two scales at the same time (the NfR was translated into Brazilian Portuguese) found a positive correlation between the Chalder fatigue scale and NfR [72].

Future research into the relationship between fatigue and PNS activation could use both HRV and pupillometry indices of SNS and PNS activation, and assess both the effects of task-evoked and daily-life fatigue [63]. Furthermore, the processes driving the PLR may be even more complex, as recent work in the fields of neuroscience and psychology has shown it to be affected by visual attention and higher-level processing [73]. The current findings suggest that PLR is also affected by more general factors like need for recovery.

## Limitations

There are several limitations of the current study that need to be mentioned. Firstly, we only measured PLR in this study. Inclusion of another independent ANS measurement like HRV would certainly be helpful to validate PLR as an ANS measurement, as well as to gain more insight into the association between fatigue and PNS. Secondly, since higher-level cognitive and attentional processing may have an impact on the PLR, it is possible that individuals with higher need for recovery found the light stimulus more annoying, and thereby perceptually more attended and subsequently evoking a stronger PLR. However, note that the light intensity applied was partly based on the results of a pilot study in which we explicitly asked participants to evaluate the comfort of the stimuli. Finally, the computer screen was not switched off during the dark adaptation period or the interval between the flashes. The background illumination of the screen may have an impact on the pupil light reflex, especially the baseline pupil diameter.

## Conclusions

The current study supports the use of a generally available display device, a computer screen, as the light source to evoke PLRs. The test-retest reliability of the set-up was tested and the results indicate good reliability on the amplitude-related parameters. The pilot study and main experiment indicate that the characteristics of the stimuli used to evoke the PLR have a strong effect on the PLR parameters. Although the associations between the parameters of the PLR as evoked by the SCREEN set-up and the parameters of the PLR evoked by a LED set-up were relatively high, the results suggest a systematic difference between the PLRs evoked by the two set-ups, with relatively large PLRs evoked by the LED system. The reliability of the PLR measurement using a simple computer screen set-up supports the potential application of PLR measurements to evaluate PNS activity in new research fields like audiology [4]. The factor analysis reveals that MCV, ACA, BPD and DV1 belong to the same factor, and this factor was likely to be related to the PNS activity. We furthermore observed that higher levels of need for recovery were associated with larger ACA, BPD and DV1 (SCREEN condition) values, suggesting larger PNS activation. This result is in line with the previous finding that people with higher levels of fatigue and need for recovery tend to show smaller pupil dilation during cognitive tasks, reflecting reduced PNS inhibition [51, 74].

## Summary recommendations for PLR measurements

- A regular PC screen can be used to evoke reliable PLRs (main experiment).
- Do not present a warning sign to announce the upcoming flash (pilot study).
- Be aware of the large effect of stimulus characteristics (color, intensity, duration) on the absolute value of the PLR parameters (pilot study).
- Do not switch between systems if one aims to compare absolute PLR values between experiments, unless the systems have been shown to generate equivalent PLR traces (main experiment).

## Supporting information

S1 Appendix Pilot study. (DOCX)

S2 Appendix Danish translated NfR questionnaire. (DOCX)

S1 Fig Baseline-corrected average PLRs of the pilot study. (TIF)

## Abbreviations

ANS: Autonomic Nervous System
PNS: Parasympathetic Nervous System
SNS: Sympathetic Nervous System
HRV: Heart Rate Variability
PLR: Pupil Light Reflex
LED: Light-emitting Diode
NfR: Need for Recovery score
ICC: Intraclass Correlation Coefficient
