# Supplementary material for: Pupil light reflex evoked by light-emitting diode and computer screen: Methodology and association with need for recovery in daily life
Source: PLoS One. 2018 Jun 13;13(6):e0197739. doi: 10.1371/journal.pone.0197739 (PMC5999086; doi:10.1371/journal.pone.0197739)
Supplement: S1 Appendix — (DOCX) [file pone.0197739.s001.docx]

# S1 Appendix – Pilot Study

## Objective

The aim of the pilot study was to develop a set-up using a computer screen to evoke and record PLRs. To test the sensitivity of the set-up, we compared the PLRs to the light stimuli characterized by different *Colors*, *Light intensities* and *Durations.*

## Methods

### Participants

The participants in the pilot study were recruited from the VU University medical center, Amsterdam via advertisements and flyers. In total, 29 (18 females) healthy adults were included in this study, with a mean age of 46.7 years (SD = 12.7). Each of the participants was a native speaker of Dutch, and without any history of neurological, psychiatric or eye diseases that might influence the pupil size. The study was approved by the medical ethical committee of the VU University medical center (register number: 2014.231), and participants were asked to sign an informed consent form before testing.

### **Pupillometry**

We used the SMI RED 500 (SensoMotoric Instruments, Berlin, Germany) remote eye tracking system to record the pupil response of both eyes with 60 Hz sampling rate (we used 120 Hz later in the experimental test) and a spatial resolution of 0.03^°^. Only data from left eye were analyzed in the current study.

### **Conditions and Procedure**

During the test, participants were seated in a comfortable chair located in a dark sound-insulated booth. The background luminance of the booth was less than 0.1 lux. A DELL P2210 (22") computer screen was placed in front of the participant, and was used as a closed-loop light source to evoke a PLR. The distance from the middle of the eyes to the center of the screen was fixed to 55 cm, with visual angles of 30° vertically and 45° horizontally. There was a small white-fixation-dot located in the center of the screen and we asked the participants to fix their eye gaze on the dot throughout the test. Eight different types of stimuli (flashes) were presented to each participant. These were based on *Color* (green (G): peak wavelength: 520 nm or Red (R): peak wavelength: 680 nm), *Light intensity* (Low (L) 3 cd/m^2^ or High (H) 10 cd/m^2^) and *Duration* (200 ms or 1000 ms). This resulted in eight conditions per participant, namely GL200, GL1000, GH200, GH1000, RL200, RL1000, RH200, RH1000. The presentation order of the eight conditions was completely randomized between participants. In each condition, the same stimulus was presented six times after a two-minute dark adaptation period. A warning sign (small-white-hollow-circle with negligible luminance change relative to the fixation mark) replaced the fixation mark a second before flash onset to warn the participants not to blink during the upcoming flash. For each flash, we recorded the pupil response for 16 seconds (15 or 15.8 seconds inter-stimulus interval depending on the duration of the flash) starting with the warning signal onset.

### **Pupil data processing**

Pupil data was processed following the same method as described in the method section of the main experiment.

### Statistical Analyses

We first examined the descriptive statistics of the PLR parameters (MCV, ACA, RCA, BPD, DV1, T75) for each of the eight conditions. Then we ran a three-way *(Color * Light Intensity * Duration)* repeated measures ANOVA on each parameter to examine the effects of within-subject factors *Color*, *Light Intensity* and *Duration* of the flash stimulus on the PLR. We expected significant within-subject effects of *Color*, *Light Intensity* and *Duration* on the selected PLR parameters.

## Results

Table A shows the descriptive statistics of the PLR parameters. The Valid N indicates the number of valid data points (participants) included in the particular condition. If there were no valid pupil traces for a given participant due to data loss and/or blinks, data for this condition were not included for this participant. In total, this occurred in 10 (out of 232) cases.

Table A. Descriptive statistics for the PLR parameters in each of the eight conditions

|  | | PNS | | | PNS + SNS | | SNS |
| --- | --- | --- | --- | --- | --- | --- | --- |
|  | Valid N | MCV (mm/s)  (SD) | ACA (mm)  (SD) | RCA (%)  (SD) | BPD (mm)  (SD) | DV1 (mm/s)  (SD) | T75 (s)  (SD) |
| RL200 | 25 | 3.53 (1.09) | 1.21 (0.32) | 25.19 (3.32) | 4.74 (0.97) | 1.79 (0.62) | 2.99 (0.70) |
| RL1000 | 29 | 3.55 (0.88) | 1.57 (0.50) | 31.98 (5.23) | 4.85 (1.13) | 2.65 (0.84) | 4.36 (0.30) |
| RH200 | 29 | 3.67 (0.88) | 1.41 (0.39) | 29.39 (3.55) | 4.78 (1.10) | 2.17 (0.72) | 3.32 (0.32) |
| RH1000 | 28 | 3.73 (0.94) | 1.80 (0.56) | 36.71 (4.64) | 4.82 (1.15) | 3.02 (0.99) | 4.18 (0.34) |
| GL200 | 28 | 3.73 (0.82) | 1.39 (0.35) | 29.95 (3.90) | 5.17 (1.15) | 2.14 (0.62) | 3.37 (1.89) |
| GL1000 | 28 | 3.75 (0.79) | 1.69 (0.45) | 33.94 (3.63) | 4.93 (1.09) | 2.75 (0.80) | 3.97 (0.28) |
| GH200 | 25 | 3.88 (0.77) | 1.49 (0.35) | 29.98 (3.53) | 4.99 (1.18) | 2.30 (0.67) | 3.55 (0.44) |
| GH1000 | 29 | 3.96 (0.78) | 1.91 (0.49) | 38.61 (3.63) | 4.92 (1.08) | 3.18 (0.90) | 4.21 (0.31) |

Valid N: number of participants with valid data for this condition; SD: standard deviation; RL200: corresponding to red light – low intensity (3 cd/m2 – 200ms duration; GH1000: corresponding to green light – high intensity (10 cd/m2) – 1000ms duration; MCV: maximum constriction velocity; ACA: absolute constriction amplitude; BPD: baseline pupil diameter; RCA: relative constriction amplitude; DV1: Dilation velocity at 1s after Maximum constriction; T75: time to reach 75% of initial resting diameter during pupillary re-dilation

S1 Fig. Baseline-corrected average PLRs of eight conditions after two minutes adaption time. A warning signal was shown during the 1-second interval prior to the flash. The baseline pupil size was determined as the mean pupil diameter recorded during 200 ms before flash onset.

S1 Fig illustrates the baseline-corrected PLR for each of the eight conditions. Results of the three-way repeated-measure ANOVA are shown in Table B. The ANOVA revealed a significant main effect of *Color* on the BPD (F (1, 20) = 14.46, p = 0.01), indicating that the BPD was higher for the green flashes than the red ones. For each of the constriction-PNS related parameters (MCV, ACA, RCA), the repeated-measure ANOVA showed significant main effects of *Color* and *Light intensity*, with relatively larger and faster PLRs for the green and higher intensity flashes as compared to red color and lower intensity stimuli. Additionally, there was a statistically significant main effect of duration on the PNS indicators ACA (F (1, 20) = 51.24, p < 0.01) and RCA (F (1, 20) = 128.37, p < 0.001). This indicates that the longer the flash, the larger the constriction observed. As for the re-dilation-SNS related parameter T75, there was a significant effect of duration (F (1, 20) = 17.85, p < 0.001), indicating that longer flashes prolonged the time to re-dilate to 75% of the original diameter (BPD). DV1, under the effect of both the PNS and SNS system, was significantly influenced by color, light intensity and duration of the flash, such that green color, high intensity and longer duration generated a faster DV1 response.

Table B Within-subject effects of *Color*, *Light Intensity* and *Duration* of the flash light on the PLR parameters

|  | PNS | | | PNS + SNS | | SNS |
| --- | --- | --- | --- | --- | --- | --- |
|  | MCV (mm/s) | ACA (mm) | RCA (%) | BPD (mm) | DV1 (mm/s) | T75 (s) |
| Color (green vs. red) | **F (1, 20) = 5.56, p = 0.03** | **F (1, 20) = 31.35, p < 0.01** | **F (1, 20) = 15.74, p = 0.001** | **F (1, 20) = 14.46, p = 0.01** | **F (1, 20) = 15.31, p = 0.001** | F (1, 20) = 0.00, p = 0.98 |
| Light Intensity (high intensity vs. low intensity) | **F (1, 20) = 22.02, p < 0.01** | **F (1, 20) = 53.57, p < 0.01** | **F (1, 20) = 192.92, p < 0.001** | F (1, 20) = 0.00, p = 0.98 | **F (1, 20) = 31.51, p < 0.001** | F (1, 20) = 0.09, p = 0.7 |
| Duration (200ms vs. 1000ms) | F (1, 20) = 1.22, p = 0.28 | **F (1, 20) = 51.24, p < 0.01** | **F (1, 20) = 128.37, p < 0.001** | F (1, 20) = 0.01, p = 0.91 | **F (1, 20) = 78.98, p < 0.001** | **F (1, 20) = 17.85, p < 0.001** |

MCV: maximum constriction velocity; ACA: absolute constriction amplitude; BPD: baseline pupil diameter; RCA: relative constriction amplitude; DV1: Dilation velocity at 1s after Maximum constriction; T75: time to reach 75% of initial resting diameter during pupillary re-dilation

Text marked bold: within-subject effect is significant at p < 0.05

## Discussion

In the pilot study, we developed and tested a PLR assessment method using a computer screen set-up generating the light stimuli. We assessed the PLR in eight conditions in which *Color*, *Light Intensity* and *Duration* of the light stimuli were manipulated. Repeated-measure ANOVAs revealed significant within-subject effects of *Color*, *Light Intensity* and *Duration* on most PLR parameters (except for BPD, MCV and T75). These results demonstrate that using a computer screen to evoke PLRs results in PLRs that sensitively reflect these different stimulus characteristics.

An interesting finding was the significant main effect of *Color* on the BPD, with PLRs to the green flash showing a larger baseline as compared to the red flash. Previous research indicates that the pre-stimuli pupil diameter (BPD) is independent from the wavelength (color) of the light stimuli [1]. The larger baseline pupil size for the PLR evoked with the green flashes may be related to a discomfort effect on the pupil size. Note that the conditions were presented in blocks - several participants indicated that the green-colored stimuli were relatively uncomfortable. This was not the case for the red stimuli. An increased pupil diameter in response to uncomfortable or painful stimuli is well documented [2, 3]. Presenting stimuli from a light source with a wide visual field (e.g. a computer screen) might increase the discomfort caused by certain wavelengths, and hence increase the baseline significantly. This has, however, never been tested formally as most studies used narrow-field light stimuli (LED) and different light stimuli [4, 5].

When inspecting the averaged PLRs in S1 Fig, we found that a pre-constriction occurred about 400 ms after the onset of the warning signal, and was evident until the warning signal offset / flash onset. We suggest that this effect may reflect that participants anticipated the flash. A similar pattern of pupil constriction has been observed during the first few seconds of picture viewing [6, 7]. Also, the pupil size decreases when participants anticipate a relatively bright stimulus, even when the stimulus intensity is kept constant [6]. Similarly, the baseline pupil size and therefore the PLRs observed in the current pilot study may have been affected by this anticipatory response, which may have subsequently reduced the sensitivity of the paradigm to measure PNS activation. Therefore, we decided to refine our PLR assessment by removing the warning signal in the later experimental test. The results of the current pilot study indicated that red stimuli are preferable to use as compared to green stimuli. Furthermore, the PLRs evoked by the red stimuli with low intensity and short duration showed good data quality. It resulted in the smallest constriction amplitude, which provides a relatively large dynamic range for between-subject and/or between-condition variations. For example, the floor effect of the pupil light reflex becomes observable when the pupil diameter is smaller than 4 mm [8], this may reduce the sensitivity of the method to detect the effect of the factors under study. Therefore, the RL200 stimuli were presented in the main experiment, using a paradigm similar to that of the pilot study, but without the warning sign.

1. Ishikawa H, Onodera A, Asakawa K, Nakadomari S, Shimizu K (2012) Effects of selective-wavelength block filters on pupillary light reflex under red and blue light stimuli. Japanese journal of ophthalmology 56: 181-186.

2. Ellermeier W, Westphal W (1995) Gender differences in pain ratings and pupil reactions to painful pressure stimuli. Pain 61: 435-439.

3. Höfle M, Kenntner-Mabiala R, Pauli P, Alpers GW (2008) You can see pain in the eye: pupillometry as an index of pain intensity under different luminance conditions. International journal of psychophysiology 70: 171-175.

4. Steinhauer SR, Condray R, Kasparek A (2000) Cognitive modulation of midbrain function: task-induced reduction of the pupillary light reflex. International Journal of Psychophysiology 39: 21-30.

5. Bitsios P, Szabadi E, Bradshaw C (1999) Comparison of the effects of venlafaxine, paroxetine and desipramine on the pupillary light reflex in man. Psychopharmacology 143: 286-292.

6. Naber M, Nakayama K (2013) Pupil responses to high-level image content. Journal of vision 13: 7-7.

7. Libby WL, Lacey BC, Lacey JI (1973) Pupillary and cardiac activity during visual attention. Psychophysiology 10: 270-294.

8. Loewenfeld IE, Newsome DA (1971) Iris mechanics I. Influence of pupil size on dynamics of pupillary movements. American journal of ophthalmology 71: 347-362.
