# Supplementary material for: Pupil light reflex evoked by light-emitting diode and computer screen: Methodology and association with need for recovery in daily life
Source: PLoS One. 2018 Jun 13;13(6):e0197739. doi: 10.1371/journal.pone.0197739 (PMC5999086; doi:10.1371/journal.pone.0197739)
Supplement: S2 Appendix — (DOCX) [file pone.0197739.s002.docx]

# S2 Appendix - Danish translated NfR scale

**Vurdering af restitutionsbehov**

**Angiv hvorvidt du er enig i følgende udsagn vedrørende din arbejdssituation**

**(er du ikke i arbejde, så svar ud fra din hverdag)**

|  | **Nej** | **Ja** |
| --- | --- | --- |
| 1. Jeg har svært ved at slappe af sidst på en arbejdsdag. | ❑ | ❑ |
| 1. Sidst på en arbejdsdag er jeg helt smadret. | ❑ | ❑ |
| 1. Mit job gør, at jeg føler mig temmelig udmattet sidst på en arbejdsdag. | ❑ | ❑ |
| 1. Efter aftensmaden føler jeg mig ofte stadig rimeligt frisk. | ❑ | ❑ |
| 1. Jeg kan som regel først slappe af på den anden fridag. | ❑ | ❑ |
| 1. Jeg skal gøre en indsats for at koncentrere mig i mine frie timer efter arbejde. | ❑ | ❑ |
| 1. Jeg har svært ved at rumme andre mennesker, når jeg selv lige er kommet hjem. | ❑ | ❑ |
| 1. Det koster mig som regel mere end en time, før jeg er kommet mig efter en arbejdsdag. | ❑ | ❑ |
| 1. Når jeg kommer hjem, skal de lige lade mig være i fred. | ❑ | ❑ |
| 1. Det sker ofte, at jeg efter en arbejdsdag er for træt til at at nå noget derhjemme den dag. | ❑ | ❑ |
| 1. Det hænder, at jeg sidst på dagen er for træt til gøre mit arbejde ordentligt. | ❑ | ❑ |
