# Supplementary figures and images for: Pupil light reflex evoked by light-emitting diode and computer screen: Methodology and association with need for recovery in daily life
Source: PLoS One. 2018 Jun 13;13(6):e0197739. doi: 10.1371/journal.pone.0197739 (PMC5999086; doi:10.1371/journal.pone.0197739)

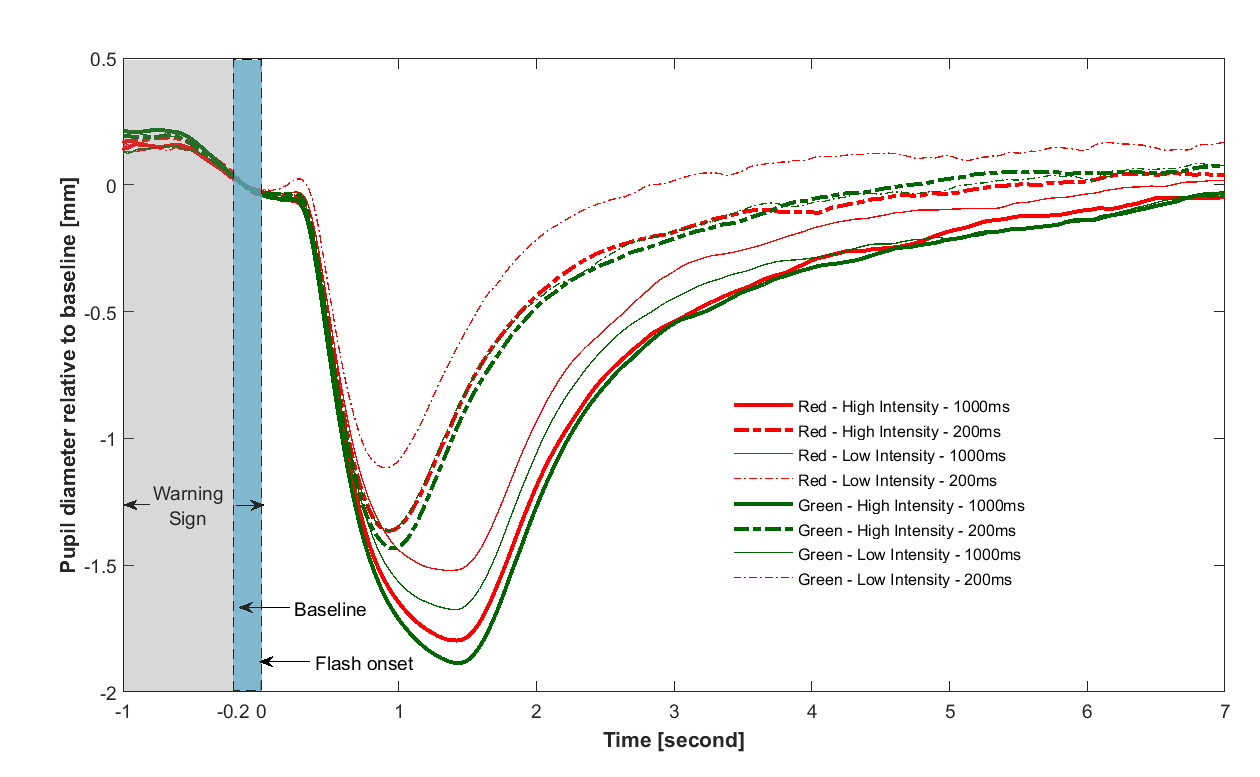

Supplement: S1 Fig — (TIF) [file pone.0197739.s003.tif]
